# Conceptualizing and Validating Marital Quality in Beijing: A Pilot Study

**DOI:** 10.1007/s11205-012-0089-6

**Published:** 2012-06-02

**Authors:** Huiping Zhang, Xiaohe Xu, Sandra K. M. Tsang

**Affiliations:** 1 Department of Social Work, The School of Sociology and Population Studies, Renmin University of China, No. 59, Zhongguancun Street, Haidian District, Beijing, People’s Republic of China; 2 Department of Sociology, University of Texas at San Antonio, One UTSA Circle, San Antonio, TX 78249-0655 USA; 3 Department of Social Work and Social Administration, The University of Hong Kong, Pokfulam Road, Hong Kong

**Keywords:** Martial quality, Chinese Marital Quality Scale, Chinese marriage, Urban China

## Abstract

Since the inception of the economic reform, marital relationship in urban China has undergone dramatic transformations. Though the burgeoning body of scholarly research has demonstrated that marital quality has increasingly become an important aspect of family life among married persons in urban China, both the conceptualization and measurement of marital quality remain underdeveloped. The purpose of this pilot study is to develop and validate a comprehensive and culturally appropriate marital quality scale, namely the Chinese Marital Quality Scale (CMQS). Results from the confirmatory factor analysis (CFA) conducted on a sample of 387 married persons from Beijing indicate that the CMQS can be conceptualized as a two-factorial and multidimensional construct, encompassing marital happiness, marital interaction, marital disagreement, marital problem, and marital instability. Additional statistical analyses also indicate that the CMQS has exhibited satisfactory reliability and concurrent validity. It is thus concluded that the CMQS is a reliable and valid instrument to measure marital quality in contemporary Beijing and possibly in other Chinese cities.

## Introduction

The landscape of urban Chinese marital relationships has undergone rapid transformations over the past six decades and marital quality has become the main indicator to measure quality of life and marital relationship in contemporary urban China (Xu 1996; Cheng et al. 2005). In traditional China, marriage was customarily viewed as an affair of two families and should be parentally arranged (Lang 1946; Xu and Whyte 1990). Under the guidance of Confucian family ethics, conjugal happiness was not only deliberately overlooked but also socially discouraged (Xu and Whyte 1990). Moreover, divorce was culturally condemned and legally monopolized by men (Sheng 2004). However, after the establishment of the People’s Republic of China, especially the enactment of the 1950 Marriage Law, the traditional institution of Chinese marriage in urban China has undergone fundamental transformations. For example, parentally arranged marriage was outlawed, love match marriage was encouraged and became popular, and conjugal relationship was emphasized (Whyte and Parish 1984; Xu and Whyte 1990). While these changes were remarkable, numerous transformations in the traditional Chinese marriage system were already under way before the communist’s takeover. The Western influences and a series of social reforms inaugurated by the nationalist government were often identified as the agents of change (Lang 1946). Since the inception of the economic reform in the late 1970s, urban Chinese marriage has undergone yet another round of profound changes. As China gradually and continually opened her door to the West in the post-Mao era, urban Chinese marriages began to converge, albeit slowly, toward their Western counterparts (Xu 1998a). Though filial obligations remain strong, emotional quality of married couples has been widely discussed and stressed (Xu 1999; Pimentel 2000; Farrer 2002). Along with increased incidences of premarital sex and premarital cohabitation (Farrer 2002), a peer-governed dating culture has emerged in major Chinese cities and the divorce rate in urban China has been steadily on a rise (Tang and Parish 2000; Zeng and Wu 2000; Wang 2001; Xu et al. 2010). As a result, urban Chinese marriage has become unprecedentedly unstable (Rich and Xu 2009). Given these remarkable changes, family scholars both inside and outside China have embarked on a new research effort to study and monitor the quality of marriages in reforming urban China.

While there has been a rich tradition in marital quality studied in the West, studies in Chinese societies in general and in urban China in particular did not emerge until fairly recently. Extant studies are limited and vary considerably in how marital quality is conceptualized and empirically measured. Some studies conceptualize marital quality as a unidimensional construct and operationalize it as a global satisfaction or happiness measure (e.g., Wu and Yi 2003; Yi and Chien 2006), whereas others conceptualize marital quality as a unidimensional construct but measure it with multiple items (e.g., Li and Chen 2002; Shek and Tsang 1993). Still others define marital quality explicitly as a multidimensional construct and measure it along multiple dimensions with multiple items (e.g., Pimentel 2000; Tang and Parish 2000; Xu 1996; Xu and Lai 2004). Even though these diverse approaches have fostered marital quality studies in Chinese societies, there are two obvious issues that need to be addressed. First, because marital quality is conceptualized and measured in so many different ways, research findings are inevitably diverse and inconsistent. Second, it is needless to say that this inconsistency makes it difficult to compare research findings across time and locales, thus preventing us from obtaining a comprehensive and accurate understanding of marital quality in urban China.

Using a sample of 387 married persons from Beijing, this study consolidates and expands on previous research by developing and validating an integrated and multidimensional Chinese Marital Quality Scale (CMQS). This endeavor is important in several different ways. First, as in other societies around the world marriage in urban China is a multifaceted social institution where dyadic or triadic interactions and exchanges take place on the daily basis. Thus, a systematic and multidimensional measurement tool is desired and needed to evaluate the quality of these interactions and exchanges. Second, marriage in urban China involves not only manifested behaviors but also subjective experiences and feelings which become increasingly important throughout the reform era (Zuo and Bian 2001; Zuo 2003). Therefore, marital quality measures must be broad enough to capture these seemingly routinized but crucial behaviors, emotions or feelings. Third, because married persons experience marriage differently across various domains of marital life (Pimentel 2000; Xu and Lai 2004), a multidimensional conceptualization can help researchers systematically investigate the determinants of the well-being of urban Chinese marriages across these diverse domains. Unfortunately, extant studies often failed to meet this research expectation. For these reasons, this study contributes to the bourgeoning body of research on marital quality in urban China.

## Marital Quality Studies in Chinese Societies

### Unidimensionality Versus Multidimensionality

The rationale for using either a unidimensional or multidimensional construct to measure and assess marital quality in Chinese societies is not well articulated (Xu 1999). In fact, with few exceptions, the decision as to what type of marital quality measures should be used is often made by convenience, such as the availability of survey data, rather than sound theoretical justifications (see Yi and Chien 2006 for example). In general, recent marital quality studies conducted in Chinese societies follow two major research traditions: the unidimensional tradition and the multidimensional tradition. While the former has been described as an individual school (Glenn 1990; Xu 1998a, b), the latter has been referred to as an adjustment school (Lewis and Spanier 1979; Xu 1998a, b). In his seminal review, Glenn made a strong plea that only marital happiness matters in marital quality study and everything else is ancillary (Glenn 1990). He insisted that marital quality can be empirically and sufficiently measured by one global measure, namely marital happiness. By contrast, the multidimensional school, such as the marital adjustment school, has gained more scholarly recognition in recent years, which routinely employs a multidimensional conceptualization and measurement. According to this line of thinking, marital quality is a hybrid concept with two latent constructs that are further indicated by five theoretically distinct dimensions. The first construct represents positive marital quality underlying marital happiness or satisfaction and positive marital interaction or togetherness, whereas the second construct signifies negative marital quality undergirding marital disagreement, problem, and instability (Johnson et al. 1986; Xu 1998a, b).

A careful review of the literature suggests that scholars who study marital quality in Chinese societies have followed disproportionately the multidimensional tradition. Though termed differently, several studies have indeed indentified two latent marital constructs with multiple measurement items, such as marital harmony and marital discord (Xu 1996; Xu and Lai 2004), marital closeness and marital disharmony (Pimentel 2000), marital satisfaction and marital conflict (Tang and Parish 2000), and marital satisfaction and marital adjustment (Shek 1995). It is imperative to note that scholars who identify with the multidimensional tradition have included at least one item that indicates marital happiness or marital satisfaction in their studies as advocated by Glenn (1990).

### Self-Reported Marital Appraisal Versus Self-Reported Marital Behavior

Another issue embedded in marital quality studies in Chinese societies is the nature of the marital quality measures. By design, scholars who have employed a unidimensional conceptualization tend to emphasize self-reported marital appraisal, such as marital satisfaction or happiness (see Wu and Yi 2003; Yi and Chien 2006). On the other hand, scholars who are identified with the multidimensional tradition tend to integrate both self-reported marital appraisal and self-reported marital behavior into their research. For example, by following Johnson et al.’s study (1986) several scholars have redefined their marital quality measures and included such key characteristics of relationship quality as companionship, communication, disagreements and/or conflicts in addition to the individual’s overall impressions or appraisals of relationship quality, namely the degree of marital satisfaction or happiness (Pimentel 2000; Shek 1995; Xu 1996; Xu and Lai 2004). The authors of this study endorse this practice and argue that marital quality measures must include both self-reported marital appraisal and self-reported marital behavior that reflect positive as well as negative aspects of marital life. It is this theoretical underpinning that drives and guides the development of the Chinese Marital Quality Scale.

### Items in the Chinese Marital Quality Scale

Johnson et al. (1986) proposed a two-factor model of marital quality with five distinct components (marital happiness, positive marital interaction, marital disagreements, marital problems and marital instability). Based on a representative national sample of 1,845 married people, they found two dimensions using the confirmatory factor analysis, with marital happiness and interaction in one and marital disagreements, problems and instability in the other. In addition, Fincham and Linfield (1997)’s clinical observation suggests that a spouse’s marital behavior is not always driven by a single undifferentiated view of his or her marriage. They conceptualized and measured marital quality as two separate dimensions rather than one, comprising positive marital quality (PMQ) and negative marital quality (NMQ), and this carving two parts of what looked like one dimension allows the study not only of happy (high in positivity and low in negativity) and unhappy (low in positivity and high in negativity) spouses but also of ambivalent spouses (high in positivity and in negativity) and indifferent spouses (low in positivity and in negativity) if necessary.

A multi-stage strategy was employed to develop the Chinese Marital Quality Scale (CMQS). In the first stage, the original questionnaire was developed by reviewing, pooling, screening, and selecting extant scales or inventories available in English (mainly including Johnson et al. (1986) and Xu (1996)’s scales). This decision was based on the following observations. First, limited marital quality studies on Chinese urban marriages since the reform tend to use measures from the United States, notably the Detroit Area Study (Blood and Wolfe 1960; Whyte 1990). Therefore, they are likely to be available in English (e.g., Pimentel 2000; Xu 1996). Second, in spite of the cultural differences between Chinese and Western marriages, the measures tailored and adopted from the Detroit Area Study have worked well in several Chinese cities, including but not limited to, Beijing and Chengdu (see Pimentel 2000; Xu 1996). It is worth noting that cultural sensitivity and properness was prioritized to guide the selection process. Culturally inappropriate measures such as “eating the main meal together and working on projects around the house” developed by Johnson et al. (1986) were screened out in the first stage. In the second stage, the synthesized English questionnaire was translated into Chinese by the first author and another Ph.D. student from the Department of Social Work and Social Administration at the University of Hong Kong independently. After the two independent translations were scrutinized and revised, they were back-translated into English by two bilingual Ph.D. students in the same University. The two English questionnaires were once again compared and examined. In the third stage, the fully developed Chinese questionnaire was presented to a panel of experts for comments and pre-tested with five married women and three married men. Finally, once the questionnaire was deemed satisfactory and finalized, it was adopted and utilized for this preliminary study in Beijing.

An ethical approval (IRB) for this study was obtained from the University of Hong Kong. An individual consent form was sent along with the questionnaire to the participants, which explained the purpose, the confidential and voluntary nature of the study. Those who agreed to participate in the study signed the consent form. The participants were informed that they would receive a token gift as an incentive if they completed and returned the questionnaire.

## Methods

### Participants

The participants in this study were recruited via a convenient sampling method in Beijing between August and September, 2009. First, 300 hundred questionnaires were distributed to 15 friends and acquaintances who were instructed to contact their friends, relatives or colleagues in Beijing to help fill out a self-administered questionnaire. Second, additional 200 questionnaires were distributed to the employees in the Adult Training Centers (ATC) in Beijing and asked them to fill out the questionnaire. On average, it took 30 min for the participants to complete the questionnaire. Out of the 500 distributed questionnaires, 387 were returned with valid information. The response rate was 77.4 %.

About 67 % of the participants were married women. On average, they were 34 years of age and had been married for 8 years. About 96 % of the participants were in their first marriage and the majority (73.4 %) reported that they had lived in a nuclear family. However, only 40 % of the respondents had a resident child who was under 18 years of age. Unlike the general population in urban China, the participants in this study were very well educated. In fact, more than 70 % of the participants received a four-year college degree or higher. Slightly more than half of the participants (53.1 %) were permanent residents in Beijing, which is consistent with Beijing’s highly mobile population characteristic. The detailed sociodemographic characteristics of the participants are presented in Table 1.

**Table 1:** Demographic characteristics of the sample (N = 387)

Variables	Percentage (%) or mean (SD)
Sex	
Male	33.4
Female	66.6
Age	
< 30	35.9
30–39	41.9
≥ 40	22.2
Years of marriage	8.19 (8.51)
First marriage	
Yes	96.0
No	4.0
Nuclear family	
Yes	73.4
No	26.6
Permanent resident in Beijing	
Yes	53.1
No	46.9
Children under 18	
Yes	40.6
No	59.4
Education attainment	
Secondary school or blow	15.3
Associate degree	14.5
Bachelor	29.4
Graduate	40.8
Employment	
Full-time	79.3
Part-time	2.8
No job or others	17.9
Household income in the past 6 months (in 10,000 Yuan)	
≤2.24	16.1
2.25–4.5	24.1
4.51–6.75	23.5
6.76–9.00	17.5
9.01–11.25	10.3
≥11.26	8.5

### CMQS Measures

As documented earlier, the CMQS was conceptualized and operationalized as a multidimensional construct which encompassed five distinctive subconstructs. To increase the reliability of each marital quality subconstruct, multiple items were utilized. By following Johnson et al.’s work (1986; see Xu 1998b as well), *marital happiness* was measured by the following 15 items. On a 4-point Likert scale ranging from 1 = *very unhappy* to 4 = *very happy*, participants were asked to rate (1) the amount of understanding received, (2) the amount of love and affection received, (3) extent to which the respondent and spouse agreed about things, (4) sexual relationship, (5) the way the spouse got along with the children (if any), (6) the spouse as a bread-winner, (7) the spouse as someone who took care of things around home, (8) the spouse as someone to do things with, (9) the spouse’s faithfulness, (10) financial situation, (11) their happiness with their home, (12) how happy the marriage was, (13) how the marriage was compared to others, (14) if the marriage was better or worse than 3 years ago, and (15) how strong feelings of love for the spouse were in the past year.

To capture the extent of *positive marital interaction* in urban Chinese marriages, 8 items were borrowed from Xu’s Chengdu study (1996). On a 4-ponit Likert scale ranging from 1 = *never* to 4 = *always*, participants were asked about the frequency of the following eight events in the past year: (1) husband and wife spent free time together, (2) husband told wife his feelings, (3) wife told husband her feelings, (4) husband showed affection, (5) wife showed affection, (6) husband showed concern, (7) wife showed concern, and (8) couples discussed big events.

*Marital disagreement* was gauged by 8 behavioral items that were used in previous studies (Johnson et al. 1986; Xu 1996, 1998b). On a 4-point Likert scale ranging from 1 = *never* to 4 = *always*, participants were asked about the frequency of marital disagreement in the past year with regard to (1) share of the housework, (2) spending money, (3) disciplining the children (if any), (4) taking care of the elderly, (5) making the opposite sex friends, (6) general issues, (7) serious quarrels in the last 2 months, and (8) slapping, hitting, punching, kicking, or throwing things at one another.

Similar to marital disagreement, the extent of *marital problems* was captured by 18 items that came from the same previous studies (Johnson et al. 1986; Xu 1996). On a 4-point Likert scale ranging from 1 = *never* to 4 = *always*, participants were asked about the frequency of marital problems in the past year with regard to: one of you (1) got angry easily, (2) had feelings that were easily hurt, (3) was jealous, (4) was domineering, (5) was critical, (6) was moody, (7) won’t talk to the other, (8) having a sexual relationship with someone else, (9) had irritating habits, (10) was not at home enough, (11) spent money foolishly, (12) drank or used drugs, (13) had been in trouble with the law, (14) refused to talk, and stomped out of the room, (15) insulted the other, (16) swore at the other, (17) kicked the other, and (18) beat the other up.

*Marital instability* was measured by 5 items from Johnson et al.’s study (1986). On a 4-point scale with 1 = *never*, 2 = *long time ago*, 3 = *in the past 3* *years*, and 4 = *recently*, participants were asked: (1) “Have you or your husband/wife ever seriously suggested the idea of divorce within the last 3 years?” (2) “Even people who get along quite well with their spouse sometimes wonder whether their marriage is working out. Have you ever thought your marriage might be in trouble?” (3) “Have you discussed the divorce with your close friend?” (4) “Has the thought of getting a divorce or separation crossed your mind in the past 3 years?” and (5) “Have you ever separated?”

In sum, positive marital interaction, marital disagreement marital problems and marital instability refer to the behavioral measures and marital happiness refers to the appraisal measures, which capture a more complete picture of marital relationships.

### Criterion Measures

To establish the CMQS’s concurrent validity, *the Chinese Kansas Marital Satisfaction Scale* (CKMSS) was included in this study. The original Kansas Marital Satisfaction Scale was developed by Schumm et al. (1986) and the Chinese version was validated in both Hong Kong (Shek et al. 1993; Shek and Tsang 1993) and Beijing (Li and Chen 2002). Respondents were asked to indicate how they had felt by rating the following 3 items on a 5-ponit Likert scale ranging from 1 = *extremely dissatisfied* to 5 = *extremely satisfied*. These items are: (1) “Generally speaking, are you satisfied with your marriage?” (2) “Are you satisfied with your spouse?” and (3) “Are you satisfied with the relationship between you and your spouse?” An index variable was computed with higher scores indicating higher levels of marital satisfaction. The reliability coefficient (Cronbach’s alpha) was 0.95.

In addition to the CKMSS, this study also incorporated the *Marital Commitment Scale* (MCS) to further enhance the CMQS’s concurrent validity. The MCS was developed by Sabatelli and Cecil-pigo (1985) and was validated among the Hong Kong Chinese population (Young 1995). On a 5-point Likert scale ranging from 1 = *totally disagree* to 5 = *totally agree*, respondents were asked to indicate the level of commitment to their marriage with reference to the following 6 statements: (1) “I feel very loyal to my partner.” (2) “It must be boring to be committed to one person.” (3) “If I had to do it all over again I would probably marry someone else.” (4) “I am willing to sacrifice for my spouse.” (5) “I can live with them, though I do not like some of my partners’ behavior.” and (6) “It does not matter if I do more for my partner than he/she for me.” After reverse-coding items 2 and 3, an index variable was constructed with higher scores reflecting higher levels of marital commitment. The reliability coefficient (Cronbach’s alpha) was 0.67.

### Statistical Analysis

In this study, five initially surmised dimensions of marital quality and their items were item-analyzed through corrected item-total correlations and exploratory factor analysis (EFA) by adopting principal component analysis with varimax rotation for factor extraction. To ensure internal consistency (i.e., reliability) of the five marital quality index variables, Cronbach’s coefficient alpha was used. For the purpose of establishing the CMQS’s concurrent validity, the bivariate relationships between marital quality measures (i.e., marital happiness, interaction, disagreement, problem, and instability) and criterion measures (i.e., CKMSS and MCS) were assessed by their Pearson Product Moment Correlations.

In order to confirm the surmised dimensionality of the CMQS, confirmatory factor analysis (CFA) in LISREL 8.7 was employed. The basic idea of CFA is to find several underlying latent factors or constructs (i.e., positive and negative aspects of marital quality) that are fewer in number than their indicator variables (i.e., five marital quality index variables: marital happiness, interaction, disagreement, problem, and instability) and can account for the intercorrelations of these observed variables. As such, the greatest advantage of using CFA over traditional EFA is the fact that CFA can effectively integrate measurement issues with model development, estimation, evaluation, and interpretation (Bollen 1989). Under the guidance of the multidimensional tradition, the one-factorial and two-factorial models were developed, estimated, and examined, respectively. When executing CFA, all missing values in the related variables were replaced with their respective means via a procedure built in LISREL (Joreskog and Sorbom 1996). Alternative methods such as listwise deletion and multiple imputation were also considered, which yielded similar, if not identical, results.

## Results

### Item Analysis

A series of the item analysis and EFA was conducted to screen out the items that either had low corrected item-total correlations or yielded negligible minor factors. As recommended by DeVellis (2003), a corrected item-total correlation of 0.45 was used as a threshold such that the items that had corrected item-total correlations lower than 0.45 were eliminated from further statistical analysis. With this in mind, several noteworthy results emerged from the item analysis and EFA. First, for measures underlying positive marital interaction and marital instability, the item analysis indicated that all corrected item-total correlations were greater than 0.45. EFA further indicated that only one factor was extracted. As a result, all items as described in the methods section under positive marital interaction and marital instability were retained. Second, for marital happiness, while all corrected item-total correlations were greater than 0.45, one minor factor underlying the respondent’s happiness with spouse as breadwinner (item 6), someone to work with (item 8), and financial situation (item 10) emerged from EFA. Consequently, these three items were excluded from further statistical analysis. Third, two items measuring marital disagreement regarding general issues (item 6) and the couple involving in slapping, hitting, punching, kicking, or throwing things at one another (item 8) were removed because their corrected item-total correlations were lower than 0.45. Finally, due to their low corrected item-total correlations, 4 items measuring marital problems (items 10, 11, 13 and 14) were deleted. In addition, EFA showed that items 8, 12, 15, 16, 17, and 18 indicated a domestic violence factor which was different from the main marital problems factor. These items were thus excluded from further analysis. It must be noted that this portion of analysis is inconsistent with a particular study conducted in Chengdu, China which used items 15, 16, 17, and 18 as indicators of marital problems (see Xu 1996). This inconsistency might have derived from the attempt to combine measures from both Johnson et al.’s study (1986) and Xu’s study (1996).

### Reliability

After the item analysis and EFA, the internal consistency of the five CMQS dimension measures were examined by using Cronbach’s coefficient alpha, inter-item correlation, and item-to-total scale correlation (see Table 2). As can be seen from the table, Cronbach’s coefficient alpha for five CMQs dimensions exhibit satisfactory reliability with Cronbach’s coefficient alpha = 0.93 for marital happiness (the number of items was reduced from 15 to 12), Cronbach’s coefficient alpha = 0.89 for positive marital interaction (all 8 items were retained), Cronbach’s coefficient alpha = 0.79 for marital disagreement (the number of items was reduced from 8 to 6), Cronbach’s coefficient alpha = 0.86 for marital problems (the number of items was reduced from 18 to 9), and Cronbach’s coefficient alpha = 0.83 for marital instability (all 5 items were retained).

**Table 2:** Reliability of CMQS

Subscales	N	r_a_	r_ii_	r_it_	Range r_it_	SE_m_
Martial happiness	319	0.929	0.519	0.700	0.522–0.830	1.44
Positive marital interaction	381	0.885	0.491	0.665	0.520–0.740	1.43
Marital disagreement	334	0.778	0.366	0.585	0.375–0.610	1.39
Marital problems	375	0.862	0.440	0.637	0.429–0.692	1.38
Marital instability	378	0.834	0.502	0.659	0.468–0.740	1.15

*Note:* *r*_*a*_ coefficient alpha reliability, *r*_*ii*_ mean inter-item correlation, *r*_*it*_ median inter-item correlation, *SE*_*m*_ standard error of measurement

### Preliminary analyses

Preliminary analyses were conducted to examine whether the five dimensions of CMQS varied according to the demographic characteristics, and the results are presented in Table 3.

**Table 3:** Descriptive characteristics of CMQS (N = 387)

Variables	Marital happiness	Positive marital interaction	Marital disagreement	Marital problems	Marital instability
	M(SD)	M(SD)	M(SD)	M(SD)	M(SD)
Sex					
1. Male	36.29 (5.09)	21.36 (3.96)	11.07 (3.17)	16.23 (3.74)	6.88 (2.52)
2. Female	35.96 (5.56)	21.20 (4.34)	10.34 (2.83)	15.85 (3.94)	7.25 (2.97)
Test statistic (F)	0.26	0.12	4.62*	0.82	1.47
Children_under_18					
1. Yes	36.02 (5.60)	20.36 (4.01)	10.75 (2.79)	16.25 (3.96)	7.56 (3.09)
2. No	36.07 (5.24)	21.85 (4.24)	10.47 (3.10)	3.80 (0.26)	6.84 (2.59)
Test statistic (F)	0.008	0.11.95***	0.74	1.20	5.99*
Education attainment					
1. Secondary or blow	34.02 (7.30)	19.42 (4.70)	11.12 (3.35)	17.20 (4.06)	8.12 (3.64)
2. Associate degree	35.38 (4.73)	19.56 (3.48)	10.42 (2.77)	16.27 (4.60)	7.23 (2.93)
3. Bachelor	36.14 (4.54)	21.71 (3.79)	10.75 (2.59)	15.65 (3.38)	6.88 (2.29)
4. Graduate	37.15 (4.83)	22.09 (4.11)	10.19 (2.98)	15.70 (3.80)	6.93 (2.74)
Test statistic (F)	4.78**	9.89***	1.56	2.62*	3.01*

* *p* < 0.05, ** *p* < 0.01, *** *p* < 0.001

Comparisons using the ANOVA test found gender difference in marital disagreement but not in other dimensions; to be specific, men reported more marital disagreement than women. There was a parenthood difference in positive marital interaction and marital instability but not in marital happiness, marital disagreement and marital problems. Parents reported lower positive marital interaction and higher marital instability than those married without children or with independent children. Differences in nearly all dimensions of CMQS except for marital disagreement were also significant. In general, high educated people (with bachelor degree and above) reported more marital happiness and positive marital interaction, and less marital problems and marital instability than those people without bachelor degree.

### Concurrent Validity

Table 4 displays the Pearson Product Moment Correlations between the CMQS dimensions (the index variables as shown in Sect. 4.2) and the two criterion measures, namely the Chinese Kansas Marital Satisfaction Scale and the Marital Commitment Scale. As anticipated, marital happiness and positive marital interaction were positively and significantly correlated with the CKMSS and MCS. Likewise, marital disagreement, marital problems, and marital instability were significantly but negatively correlated with the CKMSS and MCS. These patterned significant correlations indicated satisfactory concurrent validity for the CMQS. However, it is important to note that several correlation coefficients reported in Table 4 were below 0.3 despite the fact that they were statistically significant. It is recommended that the concurrent validity of the CMQS be further investigated in the full-scale study in the near future.

**Table 4:** Concurrent correlations of CMQS with criterion measures

	Marital happiness	Positive marital interaction	Marital disagreement	Marital problems	Marital instability	CKMSS	Marital commitment
Marital happiness	1.000						
Positive marital interaction	0.614**	1.000					
Marital disagreement	−0.291**	−0.169**	1.000				
Marital problems	−0.420**	−0.306**	0.373**	1.000			
Marital instability	−0.449**	−0.366**	0.345**	0.459**	1.000		
CKMSS	0.650**	0.516**	−0.160**	−0.165**	−0.402**	1.000	
Marital commitment	0.502**	0.402**	−0.291**	−0.291**	−0.397**	0.581**	1.000

*CKMSS* Chinese Kansas Marital Satisfaction Scale

** *p* < 0.01

### Factorial Validity

As surmised, the correlations featured in Table 4 showed two clusters. Marital happiness and positive marital interaction constituted a positive cluster whereas marital disagreement, marital problems, and marital instability formed a negative cluster. This pattern was further confirmed by the confirmatory factor analysis (CFA) where two different confirmatory factor models were developed, estimated and compared. Model 1 contained all five dimensions, namely marital happiness, interaction, disagreement, problem and instability. This model assumed that there was only one umbrella underlying construct of marital quality, embracing positive and negative measures of relationship quality (Glenn 1990). Model 2, on the other hand, presumed that there were two identifiable and separate latent constructs: one indicating the positive aspects of relationship quality and the other reflecting the negative aspects of relationship quality (Johnson et al. 1986; Xu 1996; Fincham and Linfield 1997). Because of the relatively large sample size, these two competing models were evaluated with a number of the goodness-of-fit statistics, such as the goodness of fit index (GFI), adjusted goodness of fit index (AGFI), and comparative fit index (CFI), since the traditional χ^2^ statistic is a function of sample size (Joreskog and Sorbom 1996). Nevertheless, the χ^2^ statistic along with the root mean square residual (RMR) are reported and presented in Table 5 but not emphasized.

**Table 5:** Goodness-of-fit statistics for factorial validity of CMQS

Models	χ^2^	*df*	*p*	GFI	CFI	AGFI	RMR	Result
One factor model	71.036	5	0.000	0.931	0.884	0.794	0.822	Rejected
Two factor model	25.305	4	0.000	0.974	0.957	0.904	0.583	Accepted
Difference	45.731	1	0.000					

**Fig. 1:**
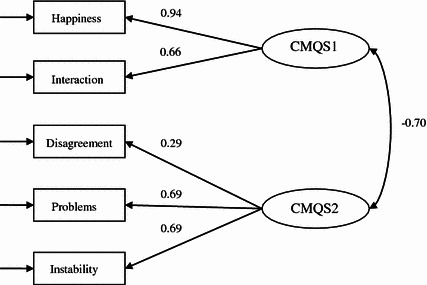
Standardized factor loadings from the two-factor CFA model

As shown in Table 5, the goodness-of-fit statistics indicate that the one-factor model did not describe the Beijing data at all well with χ^2^ = 71.036 relative to *df* = 5, GFI = 0.931, CFI = 0.884, and AGFI = 0.794. Because the two out of the three goodness-of-fit statistics were far below 0.90, the one-factor model was rejected. Turning to the two-factor model, it can be observed that the goodness-of-fit statistics were improved across the board with changes in χ^2^ = 45.731 relative to *df* = 1, which was highly statistically significant, signifying significant improvement over the one-factor model. Other goodness-of-fit statistics were also improved with GFI = 0.974, CFI = 0.957, and AGFI = 0.904. It became clear that all of these goodness-of-fit statistics were above 0.90. With these results, it was concluded that the two-factor model fit the Beijing data far better than the one-factor model and thus was accepted. As shown in Fig. 1, not only were the factor loadings of this two-factor model statistically significant (though one loading, 0.29, is relatively low), the correlation between the positive (labeled as CMQS 1) and negative (labeled as CMQS 2) marital quality factors was also statistically significant with r = −0.70. This finding is highly congruent with previous studies conducted in both North America (e.g., Johnson et al. 1986; Xu 1998a, b) and in urban China (e.g., Xu 1996).

## Discussion

Using a sample of 387 married persons from Beijing, this study confirms that marital quality is indeed a multidimensional construct that can be measured by the newly developed comprehensive CMQS. As revealed by CFA, there are two latent dimensions underlying overall marital quality in Beijing. The positive dimension, often conceptualized as marital harmony by family scholars (e.g., Pimentel 2000; Xu 1998b), can be indicated by marital happiness and positive marital interaction, whereas the negative dimension, often conceptualized as marital discord (e.g., Pimentel 2000; Xu 1998b), can be measured by marital disagreement, marital problems and marital instability. This result is remarkably consistent with previous studies.

The second worth noting finding is that the CMQS dimensions and their associated measurement items have exhibited satisfactory reliability, discriminant and concurrent validity. With few exceptions where the items must be excluded, the vast majority of items have demonstrated satisfactory levels of internal consistency. In effect, none of the reliability coefficients (Cronbach’s alpha) is less than 0.78. Regarding the preliminary analyses, the CMQS dimensions show differences in gender, dependent children and education attainment, suggesting adequate discriminant validity to some extent. As to concurrent validity, this study shows that the two previously established marital quality criterion measures, the Chinese Kansas Marital Satisfaction Scale and the Marital Commitment Scale, are significantly correlated with marital happiness, interaction, disagreement, problem, and instability in the expected directions. While nearly all previous studies conducted in urban China relied heavily on face validity, the concurrent validity test performed and reported in this study represents a new direction for future research.

Before concluding, several caveats in this study must be noted. First, because a snowball sampling method was used to recruit the participants, this study is exploratory in nature. Although a series of rigorous statistical analyses has been conducted, the results reported here cannot and should not be generalized to entire urban China. Second, for the same reason given above, the participants in this study are better educated than the general Chinese urban population. As such, care must be taken when the CMQS is used in a different urban context. Third, because of the inconsistency revealed in the item analysis pertaining to the marital problems measures, it is recommended that future research include the family violence measures for separate analysis while retaining the items originally developed and used by Johnson et al. (1986). Other eliminated items include drug use, trouble with law, and marital infidelity. Although these items are extremely sensitive in urban China, they should be retained if a large and random sample is utilized in future research. In spite of these limitations, this study is the first of its kind in developing and validating a comprehensive marital quality study instrument that is culturally appropriate. As marital quality study has gained its popularity in recent urban China, for those who are interested in the status and well-being of urban Chinese marriages the CMQS is highly recommended (the finalized items for this study are included in the appendix and the CMQS is available upon request).

## Appendix: Finalized CMQS items

### Positive Marital Quality

Marital Happiness

1. the amount of understanding received
2. the amount of love and affection received
3. extent to which the respondent and spouse agreed about things
4. sexual relationship
5. the way the spouse got along with the children (if any)
6. the spouse as someone who took care of things around home
7. the spouse’s faithfulness
8. their happiness with their home
9. how happy the marriage was
10. how the marriage was compared to others
11. if the marriage was better or worse than 3 years ago
12. how strong feelings of love for the spouse

Positive Marital Interaction

1. husband and wife spent free time together
2. husband told wife his feelings
3. wife told husband her feelings
4. husband showed affection
5. wife showed affection
6. husband showed concern
7. wife showed concern
8. couples discussed big events

### Negative Marital Quality

Marital Disagreement

1. share of the housework
2. spending money
3. disciplining the children (if any)
4. taking care of the elderly
5. making the opposite sex friends
6. serious quarrels in the last 2 months

Marital Problems

1. got angry easily
2. had feelings that were easily hurt
3. was jealous
4. was domineering
5. was critical
6. was moody
7. won’t talk to the other
8. had irritating habits

Marital Instability

1. ever seriously suggested the idea of divorce within the last 3 years
2. ever thought your marriage might be in trouble
3. discussed the divorce with your close friend
4. had the thought of getting a divorce or separation in the past 3 years
5. ever separated
